# Bitter Pills and Puffed Trials

**DOI:** 10.1371/journal.pmed.0020219

**Published:** 2005-07-26

**Authors:** Stephen Senn

**Affiliations:** **1**University of GlasgowGlasgow United Kingdom

I agree with Richard Smith [1] that something needs to be done about the reporting of pharmaceutical industry trials. Like him, I believe that the solution should include compulsory publication on the Internet of trials [2]. However, I disagree that the problem has its origin with the pharmaceutical industry; it is inherent to medical publication.

Of his eight ways of massaging data, the last five are dealt with by the International Conference on Harmonisation guidelines covering statistical principles for clinical trials (ICH E9)[3] that require prespecification of analyses. It is not possible to claim noninferiority on the basis of failure to prove a difference, and a paper describing appropriate approaches to equivalence trials that Richard Smith thought worth publishing in the *BMJ* [4] was doing no more than explaining what was common practice within the industry. The first three points are less easily policed, although choice of control group is taken extremely seriously, and, indeed, there is an appropriately entitled guideline [5] that covers this.

The problems are inherent to publication not drug regulation. An instance: the *New England Journal of Medicine* published in January 2002 a paper claiming that voriconazole is a suitable alternative to amphotericin B preparations for empirical antifungal therapy in patients with neutropenia and persistent fever [6]. However, a letter to the editor in the same issue of that journal from scientists based at the United States Food and Drug Administration [7] pointed out that the analysis presented was not what was prespecified in the protocol and that not only had voriconazole failed to demonstrate noninferiority, but it was actually statistically significantly inferior to amphotericin B. Surely, responsibility for this discrepancy cannot be laid at the door of the Food and Drug Administration, nor can it be blamed on Pfizer. Rather, the authors and the *New England Journal of Medicine* owe readers some sort of explanation.

Do the editors agree with Richard Smith (and me) that a published paper, whatever else it covers, should always identify the results of prespecified analysis, and if so, how do they check that this is so?

Thus, I agree with Richard Smith that much is wrong with the publication of clinical trials sponsored by the pharmaceutical industry. I disagree that it is a particular problem for industry trials. It is the publication process that is in need of reform, and in particular we need to scrutinize carefully the motives of authors in publishing and the standards that editors apply in deciding what gets published.

## References

[pmed-0020219-b1] Smith R (2005). Medical journals are an extension of the marketing arm of pharmaceutical companies. PLoS Med.

[pmed-0020219-b2] Senn SJ (2002). Authorship of drug industry trials. Pharm Stat.

[pmed-0020219-b3] (1999). ICH harmonised tripartite guideline. Statistical principles for clinical trials. International Conference on Harmonisation E9 Expert Working Group. Stat Med.

[pmed-0020219-b4] Jones B, Jarvis P, Lewis JA, Ebbutt AF (1996). Trials to assess equivalence: The importance of rigorous methods. BMJ.

[pmed-0020219-b5] International Conference on Harmonisation (2000). ICH harmonised tripartite guideline: Choice of control group and related issues in clinical trials E10. http://www.ich.org/MediaServer.jser?@_ID=486&@_MODE=GLB.

[pmed-0020219-b6] Walsh TJ, Pappas P, Winston DJ, Lazarus HM, Petersen F (2002). Voriconazole compared with liposomal amphotericin B for empirical antifungal therapy in patients with neutropenia and persistent fever. N Engl J Med.

[pmed-0020219-b7] Powers JH, Dixon CA, Goldberger MJ (2002). Voriconazole versus liposomal amphotericin B in patients with neutropenia and persistent fever. N Engl J Med.

